# SpiroTiger and KS Brief Stimulator: Specific Devices for Breathing and Well-Being in Post-COVID-19 Patients

**DOI:** 10.3390/jfmk9040203

**Published:** 2024-10-24

**Authors:** Maria Chiara Parisi, Donatella Di Corrado, Omar Mingrino, Caterina Crescimanno, Federica Longo, Francesco Pegreffi, Vincenzo Cristian Francavilla

**Affiliations:** 1 Department of Sport Sciences, Kore University, 94100 Enna, Italy; mariachiara.parisi@unikore.it (M.C.P.); omarmingrino@icloud.com (O.M.); federica.longo@unikorestudent.it (F.L.); 2 Department of Medicine and Surgery, Kore University, 94100 Enna, Italy; caterina.crescimanno@unikore.it (C.C.); francesco.pegreffi@unikorestudent.it (F.P.); vincenzo.francavilla@unikore.it (V.C.F.)

**Keywords:** technology, breath, posture, COVID-19

## Abstract

Background. Post-COVID-19 patients may develop impaired lung function, with reduced lung capacities and volumes, respiratory muscle weakness, and physical inactivity. The aim of this study was to determine the effects of a detailed protocol based on breathing training with two specifically designed respiratory devices (SpiroTiger and KS Brief Stimulator) in post-COVID-19 individuals. Methods. Sixteen young volunteers were randomly allocated into two groups: experimental (*n* = 8) and control (*n* = 8). The experimental group performed breathing training for 12 min two times a week for 9 months (from August 2023 to May 2024). Spirometric and baropodometric measurements were recorded every 3 months to evaluate the effects of the protocol. Results. Data analysis showed significant improvements in the forced vital capacity, peak expiratory flow indices, and postural parameters in the experimental group. Conclusions. In conclusion, based on the study findings, the specific breathing training protocol developed for post-COVID-19 patients has proven to be effective and significantly impacted the quality of breathing functions and the postural system.

## 1. Introduction

Coronavirus disease 2019 (COVID-19) is caused by the highly transmissible severe acute respiratory syndrome coronavirus 2 (SARS-CoV-2) [1]. Post-COVID-19 patients still feel persistent symptoms such as breathlessness and fatigue, and may develop an impairment of the lung function, with reduced lung capacities and volumes, respiratory muscle weakness, limitations in exercising, and a decreased functional capacity [2]. These persistent symptoms can continue for several months, leading to a condition defined as Long COVID, an often-debilitating illness that occurs in at least 10% of patients with severe acute respiratory syndrome coronavirus 2 (SARS-CoV-2) infections [3]. The World Health Organization stated that there are many actions that can be taken during the post-COVID rehabilitation process, including breathing exercises and physical exercise after discharge from the hospital [4]. The COVID-19 pandemic has brought with it a sedentary lifestyle and inactivity among the population. Therefore, working on respiratory and motor re-education is of fundamental importance not only to fight against the complications and consequences of COVID-19 but also to prevent pathologies linked to the incorrect lifestyle acquired mainly during the period of forced quarantine and inactivity due to hospitalizations for respiratory difficulties. It has been demonstrated that there is a correlation between respiratory muscle fatigue and exercise tolerance in patients exhibiting both sedentary and active lifestyles [5]. The forced and reduced physical mobility has led, in fact, many people to perceive greater fatigue, breathlessness, pain, muscular weakness, osteo-articular stiffness, and more generally a deconditioning syndrome that results in respiratory and motor alterations [6]. In this regard, some hospitals have equipped themselves to allow their patients suffering from respiratory complications due to COVID-19 (such as pneumonia) to practice respiratory and motor re-education aimed at alleviating the symptoms of the disease and physical inactivity [4]. Many studies have focused on the strong interdependence of postural control and breathing; more precisely, researchers have found that the breathing muscles are incessantly involved in the control of posture, movement, stability, cranio-cervical regions, and limbs [7,8].

Breathing muscles are involved in the breathing mechanism. The inspiratory respiratory muscles include the diaphragm, external intercostal muscles, pectoralis, serratus anterior, latissimus dorsi, serratus posterior superior, and scalene muscles. The expiratory muscles include the internal intercostal muscles, subcostal muscle, transversus thoracis muscle, anterior and lateral abdominal muscles, and serratus posterior inferior [9]. Decreases in the strength and endurance of the respiratory muscles can affect several functional and quotidian activities. Sedentary behavior is often intertwined with poor posture, which can lead to musculoskeletal imbalances and potentially impact physiological processes, limiting chest expansion and joint range of motion [10].

According to the literature, these respiratory muscles can be strengthened through resistance exercises associated with trunk stabilization exercises, developing better trunk stability and improving functions respiratory [11,12,13]. It has been shown that the most effective method to improve the lung and physical capacities of post-COVID-19 individuals involves specific and structured interventions of re-education [14,15,16,17].

Unfortunately, not many studies have been published on the topic of post-COVID-19 re-education programs. Given the lack of detailed knowledge, the aim of the present investigation was therefore to first determine the effects of a detailed protocol based on breathing training with two specifically designed respiratory devices in post-COVID-19 individuals. We selected SpiroTiger (MVM Italia S.r.l.—20020 Milan, Italy) and KS Brief Stimulator (KS International Group SRL—83013 Avellino, Italy) to train breathing muscles and improve pulmonary function. The second aim was to determine whether positive changes in breathing capacity will benefit posture and well-being, and how these could be used in future interventions aimed at public health and the prevention of possible diseases of the respiratory system. We further hypothesized that the specific properties of the devices could not only provide respiratory muscle training but also achieve an improvement in the total lung function.

## 2. Results

Sixteen young participants were randomly assigned to the experimental (*n* = 8) and control (*n* = 8) groups. The anthropometric characteristics of participants are illustrated in Table 1.

Independent *t*-tests were conducted to identify a statistically significant difference between the experimental and control groups in the postural index (Table 2).

The experimental group showed greater and significant improvements in the center-of-pressure length between T_2_ and T_3_, whereas there was no change in the control group. A paired *t*-test was conducted to detect within-group differences in the levels of spirometric parameters for each assessment during sequential testing: forced vital capacity (FVC), forced expiratory volume in the first second (FEV_1_), and peak expiratory flow (PEF) (Table 3). First, there were no significant differences in baseline characteristics between groups. Our data showed a significant increase in the experimental group compared with the control group, indicating a large effect size for the forced vital capacity at T_2_, *p* = 0.04, and T_3_, *p* = 0.02. The findings were also replicated for the forced expiratory volume in the first second at T_2_, *p* = 0.05 and T_3_, *p* = 0.03. The eta square statistic indicated a medium effect size for peak expiratory flow at T_2_, *p* = 0.05, and T_3_, *p* = 0.05.

## 3. Discussion

“Long COVID” is a term used to describe presence of various symptoms, even weeks or months after acquiring SARS-CoV-2 infection irrespective of the viral status [18]. First, the aim of the present investigation was to first determine the effects of a detailed protocol based on breathing training with two specifically designed respiratory devices in post-COVID-19 individuals. Forced vital capacity (FVC), forced expiratory volume in one second (FEV_1_), and peak expiratory flow (PEF) were used as indicators to evaluate the levels of respiratory function. The results of the present study showed significant changes in respiratory function in the experimental group compared with the control group, including improvements in forced vital capacity and expiration ability. These results are in line with those of Gloeckl et al. [19] and Liu et al. [20], who reported significant improvements in respiratory function indexes, such as FVC and FEV_1_, following respiratory training in the intervention group (individuals with long-term COVID-19). Thoracic joint mobilization promotes muscle activities for improved ventilation, also enhancing the respiratory function based on the diaphragm [21]. In this condition, the diaphragm freely works, allowing an efficient postural re-education. At this regard, the second aim of this study was to determine whether positive changes in breathing capacity would benefit posture and well-being. Various studies have demonstrated that breathing muscle training strengthens the spinal muscles, resulting in better postural control and a significantly positive impact on balance [22,23,24]. Following this line, our results showed greater and significant improvements in the center-of-pressure length in the experimental group, also achieving positive impacts on postural muscles and on the total lung function, as confirmed by spirometry.

### Limitations and New Lines of Investigation

The findings of the present study may be limited by the small sample size; in order to compare patients in groups, according to the classification of COVID-19 severity (mild, moderate, and severe), this group should be larger. Moreover, due to the small group of respondents (as the sample was less than 25), it was not possible to perform all statistical tests (e.g., a regression analysis), and this could limit the generalizability of the findings. Although the results are encouraging and relevant, further confirmatory studies are strongly recommended to assess and validate the effectiveness of the applied protocol by tracking participants over a longer period. It could provide insights into the long-term efficacy of the devices. Finally, further research is suggested to compare the SpiroTiger and KS Brief Stimulator to other breathing devices, highlighting their unique benefits and limitations.

## 4. Methods and Materials

### 4.1. Participants

Nineteen post-COVID-19 volunteers were recruited to participate in the research. The inclusion criteria were as follows: a history of COVID-19 at least 1 month prior, an absence of motor or neurological deficits, no trauma, and individuals who were available to participate in the program twice a week. The exclusion criteria were participants who had cancer or orthopedic injuries. Three participants receiving physical therapy were excluded. Sixteen young participants were randomly assigned to the experimental (*n* = 8) and control (*n* = 8) groups using the toss of a coin. The procedures were conducted according to the ethical principles stated in the Declaration of Helsinki and were performed with university ethics committee approval. The Departmental Research Committee approved the study design (approval number: 297/2023/MEDF-01/09). Before beginning the study, each participant received a full explanation of the study procedures and signed a written consent form to participate. Confidentiality of the responses was also assured.

### 4.2. Procedures

The study was conducted from August 2023 to May 2024. Participants were tested individually at four moments: T_0_ (baseline), T_1_ (after 3 months), T_2_ (after 6 months) and T_3_ (after 9 months). Every 3 months, the participants were subjected to postural stability and spirometric assessments. The postural stability assessment was performed using a baropodometric platform. All participants were placed bipedally with their bare feet side-by-side on the baropodometric platform, with each foot about 20 cm away from the other and without any type of other support.

The experimental group followed a breathing training protocol lasting 12 min twice a week; the control group observed a video clip of an individual performing the breathing function accurately and efficiently.

#### Breathing Training Protocol

The specific breathing training protocol was exclusively created for this study and included two phases: a first phase lasting for 2 min of training with SpiroTiger; and a second phase, lasting 10 min, which included the use of the KS Brief Stimulator. There were two minutes of rest between each phase.

**SpiroTiger exercises**

**Figure 1:**
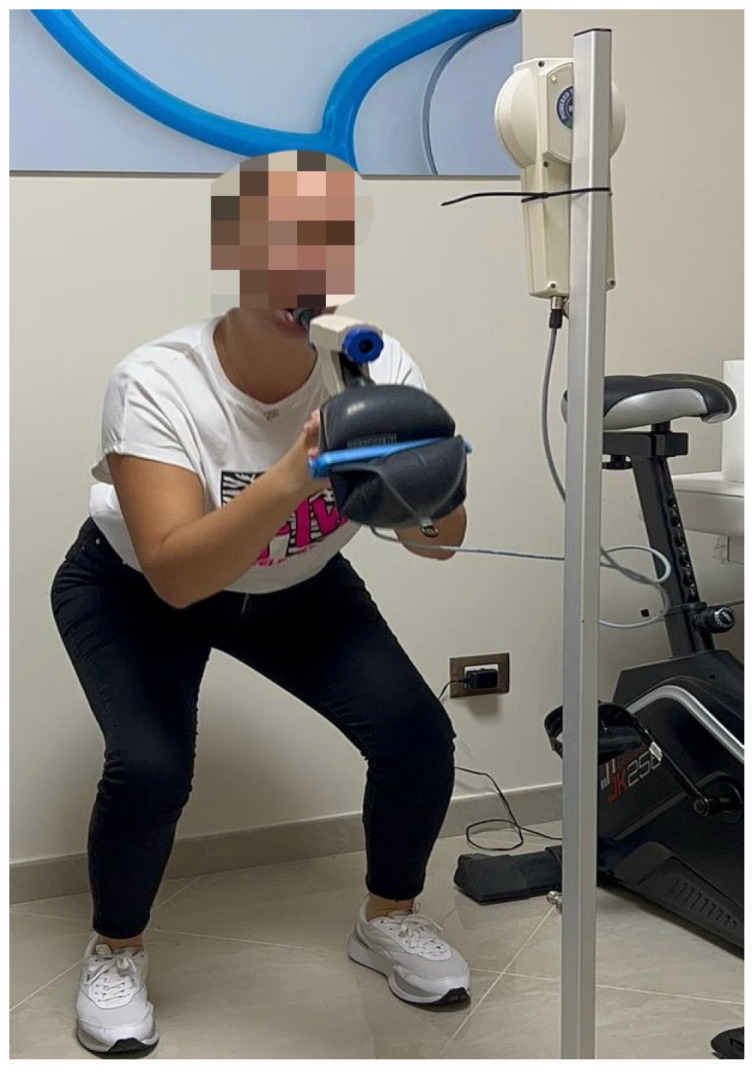
Half squat.

**Figure 2:**
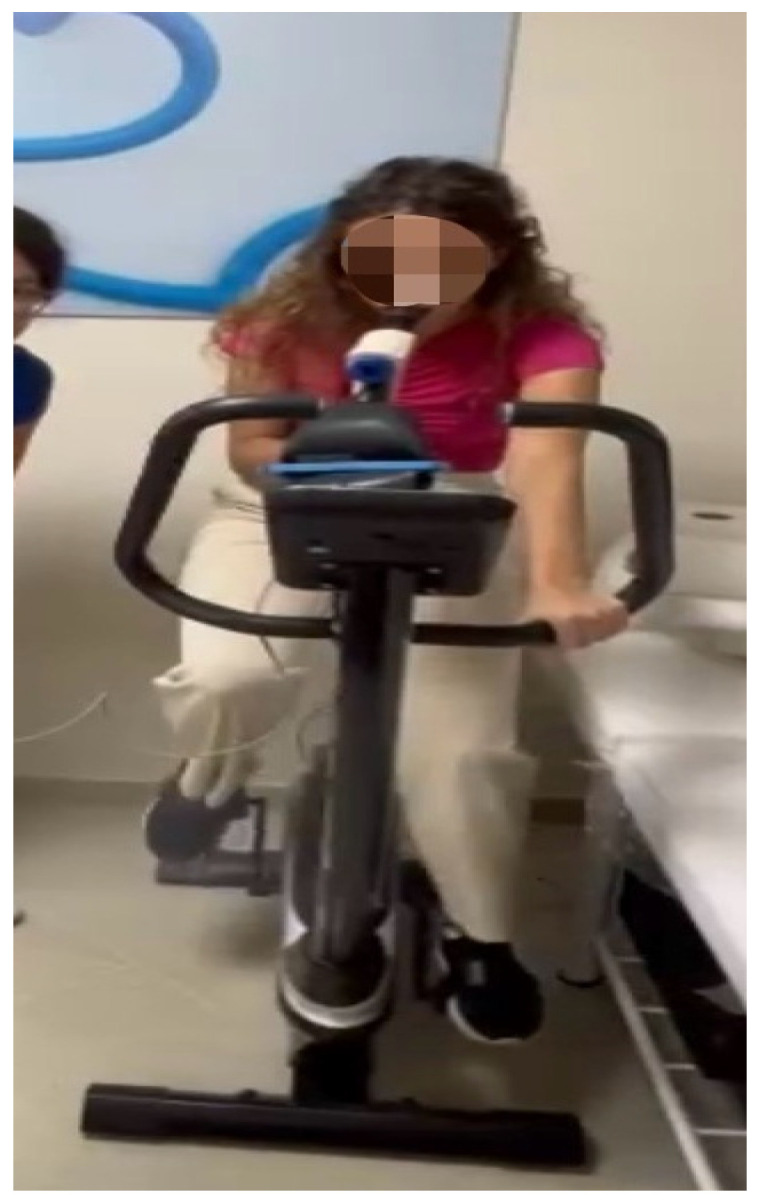
Exercise Bike.

1. Half squat (exercise duration: 1 min): the execution of this exercise included an inhalation in an upright bipedal position and a subsequent exhalation in a half-squat position, and the exercise was then repeated on the next inhalation (Figure 1).

2. Inhalation and exhalation on an exercise bike (exercise duration: 1 min): this exercise consisted of inhaling and exhaling, this time coordinating breathing while pedaling on an exercise bike at one’s own subjective pace. The size (liters) of the breathing bag used was 3.5 L. Resistance was added to make it 2.5 L for female participants (Figure 2).

**KS Brief Stimulator exercises**

**Figure 3:**
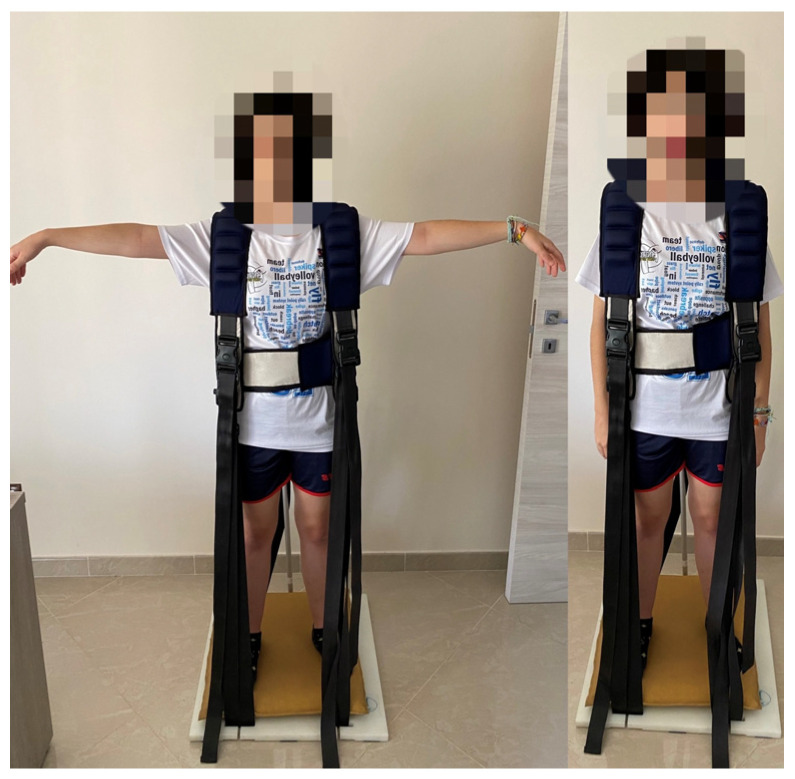
Upper Limbs 2. Flexion–extension of the head (exercise duration: 5 min): the exercise consisted of the movement of flexion of the head (forwards) on exhalation and extension of the same (backwards) on inspiration (Figure 4).

1. Abduction–adduction of the upper limbs (exercise duration: 5 min): the exercise required carrying out an abduction of the upper limbs (i.e., moving them away from the trunk) upon inspiration and a subsequent adduction of the same upon expiration (Figure 3).

### 4.3. Measurements

#### 4.3.1. Baropodometric Platform

Postural stability was measured using the FreeMed^®^ baropodometric platform and 145 FreeStep^®^ software 2.0 (Sensor Medica^®^; Guidonia Montecelio, Rome, Italy) with the following specific features: platform surface 440 × 620 mm, with an active surface of 400 × 400 mm and 8 mm thickness. According to the Romberg test, each subject was in a standing position for 51.2 s with the feet placed side-by-side, forming an angle of 30°, and both heels were 4 cm apart. The examination was conducted with open eyes. The parameter of the postural stability measurement was the center-of-pressure length (CoP_L−_mm), representing the sum of postural sway in the anteroposterior and mediolateral directions. An increase in this variable represents a decreased ability to control posture; conversely, a decrease represents an improvement [25].

#### 4.3.2. SpiroTiger

Spirometry is a procedure to measure lung function (the amount of air that enters and leaves the lungs) [26]. The first step of breathing muscle training was performed with the use of the SpiroTiger^®^ device [27]. It consists of tubing connecting a silicon breathing bag with a side port containing an opening that allows inspiration from and expiration to fresh air. During training, the breathing bag fills with expired air, characterized by elevated concentrations of carbon dioxide. When the bag is full, the valve opens and expels the remaining expired air into the environment. During inspiration, the breathing bag is first emptied completely, and then the side port valve opens, providing fresh air from the environment. The following spirometric parameters were assessed: forced vital capacity (FVC), forced expiratory volume in the first second (FEV_1_), and peak expiratory flow (PEF) [28].

#### 4.3.3. KS Brief Stimulator

The second step included the use of the KS Brief Stimulator^®^, a tool capable of specifically and permanently recovering the physiology of diaphragmatic breathing. It consists of a base with a rigid support on the ground, and two fixing systems: a set of regulating belts inserted in a linear and oblique way that move it on the chest and fix the shoulders to prevent the raising and antero-posterior expansion of the apical zone. The unique structures that can respond to the expansive force of the air are the ribs and the diaphragm, supporting their mobilization [29].

### 4.4. Statistical Analysis

The normality of the data distribution was tested using the Shapiro–Wilk test. Descriptive statistics were used to calculate the mean and standard deviation (SD). Independent *t*-tests were performed to identify statistically significant differences between the two groups in the postural index. A paired *t*-test was performed to detect within-group differences in each assessment during sequential testing. The effect size calculation (Cohen’s d) was used to evaluate the practical significance of findings, where values between 0 and 0.2 represent “very small” effects, values between 0.2 and 0.5 represent “small” effects, values between 0.5 and 0.8 represent “medium” effects, and values above 0.8 represent “large” effects [30]. All statistical analyses were processed using SPSS version 27 (SPSS Inc., Chicago, IL, USA) and presented as means ± SDs (significance level: *p* ≤ 0.05).

## 5. Conclusions

The detailed protocol based on breathing training with two specifically designed respiratory devices in post-COVID-19 individuals is highly effective at improving the functional parameters of the respiratory function. The effectiveness of the intervention had a positive impact on the functional capacity, respiratory muscle strength, and, consequently, on the postural stability, allowing for the development of a high-quality re-education protocol.

## Figures and Tables

**Figure 4:**
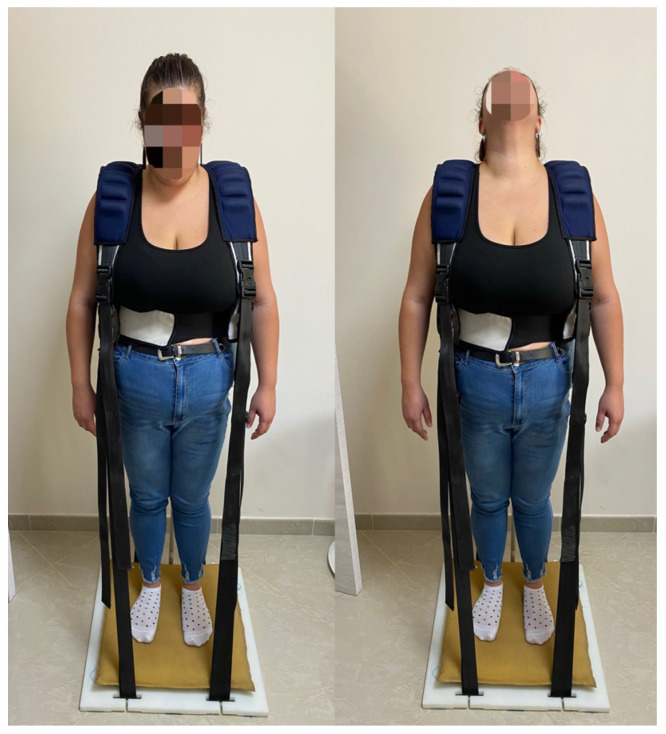
Exercise Head.

**Table 1:** Participants’ anthropometric characteristics. Means ± standard deviations.

	Age [Years]	Height [cm]	Weight [kg]	Gender
Experimental group	23 ± 2.87	170 ± 3.86	74 ± 9.29	4 m, 4 f
Control group	23.8 ± 1.48	169 ± 4.31	70 ± 8.37	4 m, 4 f
*p*	0.671	0.593	0.682	0.514

Notes: Statistically, no differences were found between these groups in terms of age, height, or weight. The groups were equally well balanced in terms of gender.

**Table 2:** Mean ± SD for the postural parameter across sessions and groups.

Postural Parameter		T_0_	T_1_	T_2_	T_3_
CoP_L−mm_	EG	279.81 ± 27.16	257.11 ± 29.13	262.47 ± 26.35 *	265.23 ± 25.14 *
	CG	282.12 ± 22.41	279.14 ± 25.63	274.14 ± 23.84	277.91 ± 25.17

Notes: * *p* < 0.05. CoP_L−mm_ = center-of-pressure length; EG = experimental group; CG = control group; T_0_ = baseline, T_1_ = after 3 months; T_2_ = after 6 months; T_3_ = after 9 months.

**Table 3:** Comparison of spirometric parameters.

		t	*p*	Mean Difference (95% CI)	Cohen’s d
FVC	T_0_	−1.24	0.26	−0.59 (−1.15~0.30)	−0.044
	T_1_	−1.99	0.08	−0.88 (−1.46~0.09)	−0.703
	T_2_	−2.43	0.04 *	−1.01 (−1.66~−0.07)	−0.861
	T_3_	−3.00	0.02 *	−1.17 (−1.92~−0.16)	−1.062
FEV_1_	T_0_	−1.31	0.23	−0.41 (−1.18~0.28)	−0.463
	T_1_	−1.56	0.16	−0.49 (−1.28~0.21)	−0.550
	T_2_	−2.19	0.05 *	−0.64 (−1.55~0.05)	−0.786
	T_3_	−2.56	0.03 *	−0.71 (−1.72~−0.04)	−0.904
PEF	T_0_	−2.03	0.08	−1.46 (−1.52~0.08)	−0.718
	T_1_	−2.16	0.06	−1.66 (−1.54~0.05)	−0.765
	T_2_	−2.24	0.05 *	−1.74 (−1.48~0.03)	−0.793
	T_3_	−2.11	0.05 *	−1.93 (−1.42~0.06)	−0.795

Notes: * *p* < 0.05. FVC = forced vital capacity, FEV_1_ = forced expiratory volume in the first second, PEF = peak expiratory flow; T_0_ = baseline, T_1_ = after 3 months; T_2_ = after 6 months; T_3_ = after 9 months.

## Data Availability

The data that support the findings of this study are available from the corresponding author (D.D.C.) upon reasonable request.
